# Single-Cell Multi-Omics Profiling of Human Septal Myectomy Tissue: Toward Precision Medicine in Obstructive Hypertrophic Cardiomyopathy

**DOI:** 10.3390/jpm16020088

**Published:** 2026-02-04

**Authors:** Quynh Nguyen, Jeremy Parker, Amrit Singh, Ying Wang, Jamil Bashir, Zachary Laksman

**Affiliations:** 1 Division of Cardiac Surgery, Department of Surgery, University of British Columbia, Vancouver, BC V6Z 1Y6, Canada; 2 School of Biomedical Engineering, University of British Columbia, Vancouver, BC V6T 1Z4, Canada; 3 Centre for Heart Lung Innovation, University of British Columbia, Vancouver, BC V6Z 1Y6, Canadaying.wang@hli.ubc.ca (Y.W.); 4 Providence Research, Providence Health Care, Vancouver, BC V6Z 1Y6, Canada; 5 Division of Cardiology, Department of Medicine, University of British Columbia, Vancouver, BC V6Z 1Y6, Canada; 6 Department of Anesthesiology, Pharmacology and Therapeutics, University of British Columbia, Vancouver, BC V5Z 1M9, Canada; 7 Department of Pathology and Laboratory Medicine, University of British Columbia, Vancouver, BC V5Z 1M9, Canada; 8 St. Paul’s Hospital, Vancouver, BC V6Z 1Y6, Canada

**Keywords:** Hypertrophic cardiomyopathy, septal myectomy, single-cell RNA sequencing, single-nucleus RNA sequencing, spatial transcriptomics, multi-omics

## Abstract

Hypertrophic cardiomyopathy (HCM) is an inherited cardiac disorder most commonly caused by pathogenic variants in sarcomeric genes, yet many patients remain genotype-negative and the mechanisms linking genetic alterations to disease pathology are not fully understood. Traditional bulk analyses have provided limited insight into the cellular and molecular changes that drive disease progression. Recent advances in single-cell and spatial multi-omics technologies now allow detailed characterization of cell type-specific transcriptional programs, signaling pathways, and tissue remodeling within the human myocardium. These approaches have begun to redefine HCM as a complex, multicellular disease rather than a purely sarcomeric disorder. This review summarizes current single-cell and spatial transcriptomic studies of human septal myectomy tissue, outlines their major findings and limitations, and discusses how these data may inform the development of precision medicine strategies in obstructive HCM.

## 1. Introduction

Hypertrophic cardiomyopathy (HCM) is an inherited condition characterized by left ventricular hypertrophy that cannot be explained by other cardiac, systemic, or metabolic conditions [1]. It affects approximately 1 in 200 to 500 individuals and is a major cause of heart failure, arrhythmia, and sudden cardiac death [2].

HCM shows significant genetic heterogeneity. It is typically inherited in an autosomal dominant pattern with incomplete penetrance and variable expressivity [3]. Pathogenic variants in sarcomeric genes encoding components of the cardiac contractile apparatus represent the most common cause. Genetic testing identifies mutations in 30–60% of clinically diagnosed cases, with MYBPC3 and MYH7 together accounting for about 70% of variant-positive patients [4]. To date, more than 1500 distinct sarcomeric variants have been described, while approximately 40% of patients have no identifiable genetic mutation [5].

Current therapies for HCM largely target downstream sarcolemmal and hemodynamic mechanisms rather than the underlying molecular defect [6]. Beta-adrenergic receptor blockers and non-dihydropyridine calcium channel blockers reduce hypercontractility by modulating the excitation-contraction process, while septal reduction therapies are reserved for patients with obstructive disease. Implantable cardioverter-defibrillators are used in those with high-risk features for sudden cardiac death [7]. Approximately 1–2% of patients with progressive, medically refractory, end-stage systolic dysfunction in the absence of outflow obstruction ultimately require heart transplantation.

Recently, precision therapy has emerged with the introduction of mavacamten [8,9]. This cardiac myosin ATPase inhibitor directly reduces excessive actin—myosin interactions which is a key downstream contractile abnormality observed in many patients with HCM, particularly those with sarcomeric gene mutations. By modulating sarcomere function, mavacamten normalizes myocardial contraction and relaxation and offers the first targeted treatment that addresses the disease mechanism. The genetic and phenotypic variability among HCM patients highlights the need to understand the molecular basis of HCM, which will guide the development of additional mechanism-based therapies.

In recent years, single-cell technologies have transformed cardiovascular research by enabling gene expression and molecular profiling at single-cell resolution, along with spatial mapping of cell–cell interactions [10,11,12,13]. These approaches provide unprecedented insights into the cellular composition and molecular architecture of human tissues. In HCM, septal myectomy specimens from septal reduction therapy represent a uniquely accessible source of diseased human myocardium and have become an invaluable resource for single-cell and multi-omics studies.

This review summarizes the genetic basis of HCM, provides an overview of emerging single-cell and multi-omics technologies applied to septal myectomy tissue, and discusses how these findings can inform personalized approaches to HCM therapy. Septal myectomy specimens are particularly informative because they are obtained from patients with obstructive HCM at a relatively earlier stage of disease before the extensive remodeling seen in end-stage heart failure with explanted heart specimens prior to transplantation.

## 2. Genetics of HCM

The first gene implicated in HCM was MYH7, which encodes β-myosin heavy chain [14]. Subsequent discoveries of pathogenic variants in other sarcomeric genes such as MYBPC3, TNNT2, and TNNI3 established HCM as a disease of the sarcomere [15,16]. Among these, MYH7 and MYBPC3 are the most frequently affected genes, together accounting for the majority of genotype-positive cases [17].

Mutations in MYH7 are primarily missense variants caused by single-nucleotide substitutions leading to amino acid changes [14,18]. These often exert a dominant-negative effect, where the mutant protein integrates into the sarcomere and interferes with normal filament assembly and contractile function. In contrast, MYBPC3 mutations are often truncating including nonsense, frameshift, or splice-site variants that produce premature stop codons and result in haploinsufficiency due to reduced functional protein expression [19].

The eight core sarcomeric genes (MYBPC3, MYH7, TNNT2, TNNI3, TPM1, MYL2, MYL3 and ACTC1) account for over 90% of genotype-positive cases and remain those with the strongest evidence for pathogenicity [20]. With advances in high-throughput sequencing, additional genes with moderate to strong associations have been identified, including JPH2, CSRP3, FHOD3, ALPK3, TRIM63, PLN, and FLNC [21,22,23,24,25,26]. Some candidate genes encoding Z-disk and M-band proteins have been proposed, though their causal roles remain uncertain [21].

HCM is classically inherited in an autosomal dominant manner with incomplete penetrance and variable expressivity, but recessive and X-linked inheritance have been described in rare cases [22,27]. Long-term follow-up studies report approximately 50% penetrance among sarcomere-positive carriers, with male sex and ECG abnormalities predicting higher penetrance [28]. Population-based cohorts show lower penetrance around 18% in the UK Biobank and roughly 11% in meta-analyses of incidentally identified carriers [29,30]. Regardless, 40–60% of clinically diagnosed HCM patients remain genotype-negative, suggesting roles for polygenic inheritance and non-genetic or environmental modifiers.

Although key causal genes have been identified, how these mutations lead to the cellular, molecular, and structural changes observed in HCM is not fully understood. Multi-omics studies, particularly at the single-cell level, are beginning to address this gap.

## 3. Multi-Omics Technology

Bulk RNA sequencing has provided important insights into gene expression in HCM but is limited by its averaging of signals across diverse cell types, which masks cell type specific changes. Single-cell technologies overcome this limitation by profiling individual cells or nuclei, allowing analysis of cellular diversity, cell state-specific gene expression, intercellular communication, and transcriptional dynamics through trajectory analysis [11]. While these approaches reveal novel molecular signatures within distinct cell types, they do not preserve the spatial relationships between cells. To address this limitation, spatial transcriptomic techniques have been developed to reconstruct gene expression in tissue context, providing information on how different cell types interact within their native microenvironment [13]. Detailed technical reviews of these methods are available in previous review papers. Here, we provide a focused overview of single-cell RNA sequencing (scRNA-seq) and spatial transcriptomics, which are currently the most widely used single-cell based approaches in HCM research.

### 3.1. Single-Cell and Single-Nucleus RNA Sequencing

The principle behind single-cell RNA sequencing relies on two key components: the generation of a high-quality single-cell suspension and the use of microfluidic technology to isolate individual cells into droplets for barcoding and sequencing [31]. In this system, each droplet contains a single cell or nucleus along with reagents for reverse transcription, thus enabling capture of cell-specific transcriptomes. The resulting libraries are then sequenced to produce expression profiles for thousands of individual cells.

For cardiac tissue, generating a viable single-cell suspension is particularly challenging. The myocardium has a dense collagen matrix, and adult cardiomyocytes are large, elongated, and fragile, making them highly susceptible to damage during enzymatic and mechanical dissociation [12,32,33]. As a result, most cardiac studies use single-nucleus RNA sequencing (snRNA-seq) rather than whole-cell approaches. This method isolates nuclei from frozen or fixed tissue, allowing transcriptional profiling of heart samples while minimizing issues with dissociation. However, snRNA-seq generally detects fewer total genes and protein-coding transcripts per cell compared with scRNA-seq and is enriched for nuclear-retained and nascent transcripts, whereas scRNA-seq captures a broader cytoplasmic transcriptome.

After sequencing, data processing typically follows a standardized pipeline [34,35]. Quality control involves removing ambient RNA, low-quality nuclei and multiplets, followed by normalization and batch correction to account for technical variation. Dimensionality reduction and clustering are then performed to group cells or nuclei based on transcriptional similarity, allowing cell type-specific identification through marker gene expression.

Single-cell and single-nucleus RNA sequencing can uncover transcriptional heterogeneity within cell populations and identify previously unrecognized cellular subtypes or activation states [11,12,32,36,37]. Differential gene expression and pathway analyses provide insight into the biological processes active within each cell type, while ligand-receptor interaction analysis reveals patterns of intercellular communication. Finally, trajectory inference methods model dynamic processes such as differentiation, stress response, or disease progression by ordering cell states along a computational pseudotime axis according to similarities in gene expression profiles.

### 3.2. Spatial Transcriptomics

While single-cell RNA sequencing provides detailed information on cell type specific gene expression, it requires tissue dissociation and therefore loses the spatial context of cells within their native environment. Spatial transcriptomics addresses this limitation by mapping gene expression back to its original tissue location. This allows for an investigation of how different cell types are organized and interact within intact tissue architecture. This spatial information is particularly important in HCM, where key pathological features such as myocyte disarray, regional fibrosis, and microvascular remodeling are spatially heterogeneous rather than uniformly distributed. Linking transcriptional states to specific histological regions enables direct association between molecular programs and disease-relevant tissue architecture, which cannot be inferred from dissociated single-cell data alone.

Spatial transcriptomic techniques can be broadly divided into sequencing-based and imaging-based approaches, each with distinct advantages and trade-offs [10,38,39,40,41]. Sequencing-based methods, such as 10× Genomics Visium, use spatially barcoded capture probes placed on a slide [42,43]. Transcripts from the overlying tissue bind to these probes, are reverse-transcribed, and sequenced using next-generation sequencing. The spatial barcodes allow each transcript to be mapped to a defined coordinate, producing a spatially resolved gene expression matrix that can be aligned with histological images. Imaging-based methods, such as MERFISH or seqFISH, visualize RNA molecules directly in situ using fluorescent probes and microscopy [44,45,46]. These approaches achieve higher spatial resolution but are often limited to predefined gene panels and require more complex imaging workflows.

Data analysis in spatial transcriptomics generally consists of pre-processing and downstream analysis [39,47,48,49]. Pre-processing converts raw imaging or sequencing data into a spatially resolved gene expression matrix. For imaging-based approaches, this involves identifying the location and expression level of RNA molecules within the tissue image and correcting for background or noise. For sequencing-based methods, pre-processing begins with tissue image registration and division of the capture area into spatial spots, followed by alignment of sequenced reads to a reference genome. The spatial coordinates from the image and the gene expression data are then combined to produce a spatially indexed transcript count matrix. These pre-processing steps are then followed by normalization and dimensionality reduction to identify spatial domains with distinct molecular signatures. Integration with single-cell or single-nucleus RNA sequencing data enables cell type deconvolution, allowing each spatial spot to be annotated based on its likely cellular composition and revealing how molecular programs are organized across the tissue.

Spatial transcriptomics provides insights that cannot be obtained from dissociated single-cell data alone. It reveals spatial gene expression gradients, cellular neighborhoods, and cell–cell interaction networks within the structural framework of the tissue [50,51,52,53,54]. When integrated with single-cell RNA sequencing, spatial data validate transcriptional findings and link molecular signatures to tissue architecture, enabling a more complete understanding of disease processes such as fibrosis, inflammation, and myocardial remodeling in HCM [55,56].

Together, single-cell and spatial transcriptomic approaches provide complementary insights into the cellular and molecular architecture of the myocardium. Single-cell RNA sequencing defines the transcriptional identity and state of individual cell types and is commonly used as a reference for deconvoluting spatial transcriptomic data, while spatial transcriptomics place these profiles within their histological and microanatomical context. In healthy human septal tissue, these techniques have revealed substantial cellular heterogeneity, including diverse cardiomyocyte, fibroblast, endothelial, and immune subpopulations organized in distinct spatial domains [57,58]. Understanding this baseline organization is essential for interpreting disease-associated changes. Integrating single-cell and spatial data in hypertrophic cardiomyopathy has begun to uncover how this normal architecture is disrupted, revealing altered cell–cell interactions, signaling pathways, and region-specific remodeling that underlie septal hypertrophy. The following section summarizes key findings from recent single-cell and spatial multi-omics studies of both healthy and HCM septal tissue.

## 4. Heterogeneity in Healthy Human Septal Tissue

A single-nucleus RNA sequencing study by Larson and Chin profiled 24,858 nuclei from four adult human interventricular septal samples and identified 23 distinct cell populations [58]. Approximately one-third of nuclei were cardiomyocytes, one-third were fibroblasts, and about one-fifth were immune cells, with smaller populations of smooth muscle cells, pericytes, endothelial cells, neuronal, lymphatic, and stromal cells. Within the cardiomyocyte compartment, five transcriptionally distinct subpopulations were observed that differed in pathways related to oxidative phosphorylation and protein synthesis. Six fibroblast (FB) subpopulations were identified, with notable heterogeneity in extracellular matrix gene expression; FB1 and FB3 expressed minimal collagen, while FB5 showed the highest collagen expression. Some fibroblast states (e.g., FB2 and FB5) also demonstrated increased activation of proinflammatory and signaling pathways. Together, these findings highlight the substantial cellular and functional diversity present within the healthy human septum.

In addition to septum-focused profiling studies, a comprehensive single-cell and single-nucleus transcriptomic atlas of the adult human heart has characterized cellular diversity across six anatomic regions, including the interventricular septum [57]. This dataset profiled over 480,000 nuclei and identified major cardiac cell types such as cardiomyocytes, fibroblasts, endothelial cells, mural cells, immune cells, adipocytes, mesothelial cells, and neuronal subtypes, demonstrating that the healthy adult heart is composed of multiple transcriptionally distinct populations. Within the ventricular regions (apex, septum, and left ventricle), approximately half of all nuclei were ventricular cardiomyocytes, with the remainder composed of mural cells, fibroblasts, endothelial cells, and immune cells. Five ventricular cardiomyocyte subpopulations were identified, differing in metabolic, stress-response, contractile, and conduction-associated gene programs. Similar heterogeneity was present within fibroblasts, vascular cells, and immune populations, reflecting regional specialization within the normal myocardium. Although this atlas was not septum-specific, it includes septal nuclei and provides an essential reference framework for baseline ventricular cardiomyocyte and stromal diversity, against which disease-associated remodeling in HCM can be interpreted.

## 5. Insight from Multi-Omic Studies

### 5.1. Single-Nucleus RNA Sequencing Studies

A single-nucleus RNA sequencing study by Larson et al. profiled septal myectomy tissue from nine patients with HCM and compared this with donor control hearts (Table 1) [59]. The final dataset included over 109,000 nuclei from HCM hearts and approximately 25,000 nuclei from donor hearts, classified into 10 major cell types and 34 transcriptionally distinct populations. Thirteen cardiomyocyte subpopulations and eight fibroblast subpopulations were identified, along with endothelial, lymphatic, smooth muscle, pericyte, neuronal, stromal, and immune cell populations. Cardiomyocytes represented a higher proportion of nuclei in HCM compared to controls. Trajectory analysis showed that the relationships between cell subtypes were preserved between normal and HCM tissue, but differential expression along the trajectories revealed disease-associated changes in gene activity.

Differential gene expression analysis identified 28 genes with spatially coherent differences between normal and HCM tissue, many of which were sarcomere-related or extracellular matrix associated. Sarcomeric genes such as ACTA1, ACTC1, MYL7, MYL2, TNNI2, and TNNI3 were upregulated in HCM across multiple cell populations, supporting known alterations in contractile machinery. Several extracellular matrix genes, including COL1A1, COL6A1, COL6A2, CYR61, and POSTN, were down regulated in fibroblasts, suggesting remodeling of ECM composition. Gene Ontology analysis showed increased enrichment for molecular functions involving actin binding, filament sliding, contraction, and sarcomeric processes in HCM, with decreased enrichment for ECM-related functions, TGF-β signaling responses, steroid hormone pathways, immune response regulation, and ECM organization.

Ligand-receptor analysis demonstrated a global reduction in intercellular communication in HCM tissue compared with normal donor hearts, with more than a two-fold decrease in total ligand-receptor pairs (546 vs. 1138). Most ligand molecular functions were markedly reduced, including extracellular matrix structural constituents, tensile strength ECM proteins, growth factor binding, protease binding, platelet-derived growth factor binding, and several of these functions were completely lost across multiple cell types in HCM. Despite this overall reduction, there were specific gains in communication involving integrin signaling. In HCM, lymphocytes acquired ITGB1 receptor expression, enabling new interactions with fibroblast-derived COL1A1 and COL1A2 ligands. Fibroblast subtypes in HCM displayed a striking loss of broadcasting ligands overall, particularly fibroblast cluster 4, whose lost ligands were enriched for integrin binding and ECM structural functions. However, communication from fibroblast clusters 2–5 to cardiomyocyte clusters 1, 2, and 13 increased 3.2-fold in HCM, driven by ITGB1 receptor expression in these cardiomyocyte clusters and expression of multiple cognate ligands in HCM fibroblast clusters that were not present in controls. Additionally, communication from cardiomyocyte cluster 8 to other cardiomyocyte clusters increased via the same ITGB1 axis. Together, these findings indicate that while global cell–cell communication is reduced in HCM, there is selective enhancement of integrin-mediated signaling between fibroblasts, lymphocytes, and specific cardiomyocyte states, implicating altered ECM-integrin pathways as a central disease mechanism.

### 5.2. Single-Cell RNA Sequencing Studies

While single-nucleus RNA sequencing studies have provided important insights into global cellular heterogeneity and intercellular communication in HCM, they primarily capture nuclear transcripts. In contrast, single-cell RNA sequencing of isolated cardiomyocytes allows for higher resolution characterization of cytoplasmic transcripts and cell state diversity within the cardiomyocyte compartment itself. These complementary approaches have revealed distinct and sometimes non-overlapping aspects of HCM biology, underscoring that disease mechanisms inferred from nuclei-level datasets may not fully recapitulate cardiomyocyte-specific transcriptional remodeling. The following studies illustrate how single-cell profiling of cardiomyocytes refines our understanding of hypertrophic remodeling at the single-cell level.

A single-cell study by Wehrens et al. profiled isolated cardiomyocytes from five HCM septal myectomy samples and compared them with previously published control single-cell cardiomyocyte datasets [60]. Six cardiomyocyte subpopulations were identified within HCM samples. Pathway analysis demonstrated that clusters 1 and 2 were enriched for sarcomere-related gene programs, whereas clusters 3 and 4 were enriched for signaling and metabolic processes. NPPA expression was not uniformly elevated across all disease cardiomyocytes, but instead was concentrated in a discrete subpopulation enriched in HCM and located adjacent to fibrotic regions on histological validation. Gene-gene correlation analysis revealed genes with strong positive correlation to NPPA, including NPPB, ACTC1, MYL4, XIRP1 and RTN4, and genes with negative correlation such as XIRP2, which clustered in a distinct cardiomyocyte state. XIRP2-correlated genes such as CMYA5, ZNF106, and MAP4 were also enriched in HCM cardiomyocytes, suggesting additional disease-associated substructure.

Transcription factor regulatory network inference using SCENIC was performed for each patient independently, and consensus filtering based on shared presence and consistent target gene sets identified 22 regulons reproducibly active in HCM cardiomyocytes. Gene Ontology analysis of these regulons revealed functional associations with metabolic processes, sarcomere/cytoskeletal organization, and membrane targeting, consistent with multiple pathogenic axes in HCM. Independent of TF expression, gene-gene correlation analysis identified five shared co-expression modules conserved across patients, with modules related to mitochondrial ATP processes, sarcomere structure, and calcium handling. Using index-sorting flow cytometry to link single-cell transcriptomes with FSC-A as a proxy for cell size, the authors showed that several module 2 genes were strongly correlated with cardiomyocyte hypertrophy. Correlation patterns were confirmed in separately acquired HCM myectomy RNA datasets, supporting module 2 as a disease-relevant hypertrophy-associated transcriptional signature.

Another study performed high-resolution single-cell RNA sequencing specifically on cardiomyocytes isolated from septal myectomy samples of patients with HCM (n = 7) and compared them with controls (n = 2) [61]. This dataset included 1971 cardiomyocytes and identified 130 upregulated and 61 downregulated genes in HCM. Upregulated gene programs were enriched for pathways related to heart development, muscle contraction, actin cytoskeleton organization, and cell adhesion, whereas downregulated programs were associated with oxidative phosphorylation, ribosome biogenesis, and cell maturation, consistent with a metabolic shift and a more fetal-like transcriptional state in HCM cardiomyocytes. ACE2 and multiple SARS-CoV-2 infection pathway genes were also upregulated in HCM.

Regulatory network analysis using SCENIC demonstrated a predominance of transcription factor activation in HCM cardiomyocytes, including increased activity of KLF5, ETV1, and TCF4, whereas factors associated with immune regulation such as CEBPB and CEBPD were reduced. To increase statistical power, the single-cell dataset was combined with prior single-cell and single-nucleus studies, generating a meta-dataset of over 65,000 HCM cardiomyocytes and 94,000 control cardiomyocytes. After stringent filtering, 653 upregulated and 185 downregulated genes were identified as signature markers of HCM, including conserved modules enriched for muscle structural development, MAPK signaling, and calcium ion import dysregulation.

Clustering analysis revealed five transcriptionally distinct cardiomyocyte states in HCM compared with three in controls, indicating increased heterogeneity in disease. Individual HCM clusters were enriched for pathways related to hypertrophic signaling, viral interaction pathways, growth factor signaling, energy homeostasis, and chromatin regulation. Importantly, several extracellular matrix genes such as LUM, DCN, FN1, CTGF, and COL1A2 were upregulated across HCM cardiomyocyte subpopulations, suggesting that cardiomyocytes themselves contribute to extracellular matrix remodeling in HCM rather than fibroblasts alone.

Chen et al. profiled cardiomyocytes isolated from septal myectomy tissue using single-cell tagged reverse transcription sequencing and identified three major cardiomyocyte subpopulations [62]. Co-expression module analysis defined five gene modules representing metabolic processes (M1), muscle structure development (M2), ATP metabolism (M3), cell cycle (M4), and cell differentiation (M5). Modules related to metabolism (M1) and muscle structural processes (M2) were most enriched in one cardiomyocyte cluster, whereas modules related to oxidative phosphorylation and ATP synthesis (M3) were enriched in a second cluster. A third cluster showed intermediate expression patterns. Correlating module expression with cardiomyocyte surface area demonstrated that the ATP metabolism module (M3) was most strongly associated with increased cell size in HCM cardiomyocytes. Hub genes in this module, including COX7B, COX5B, COX6C, ATP5ME, and other Complex IV components, were significantly upregulated in hypertrophic cardiomyocytes compared with controls. COX7B expression was further examined across HCM stage: it was increased in early and mid-stage hypertrophy but decreased in advanced HCM with reduced cardiac function and end-stage heart failure, suggesting stage-dependent regulation. In a pressure overload mouse model, knockdown of Cox7b accelerated heart failure progression, whereas overexpression partially restored cardiac function. This study supports a role for mitochondrial Complex IV remodeling in cardiomyocyte hypertrophy and identifies COX7B as a potential early-stage compensatory target.

### 5.3. Spatial Transcriptomics Studies

Liu et al. combined single-nucleus RNA sequencing with spatial transcriptomics to investigate lineage-specific regulatory changes in HCM [55]. Single-nucleus profiling identified nine major cardiac lineages and revealed marked shifts in cellular composition, including expansion of vascular-related cells (vascular endothelial cells, pericytes, and smooth muscle cells) and contraction of cardiomyocytes and fibroblasts, consistent with increased cell loss in HCM. Cardiomyocytes segregated into two main states: CM1, marked by FGF12 and interpreted as a compensated or homeostatic hypertrophy state, and CM2, marked by NPPB and ACTA1, representing a fetal-like failing cardiomyocyte state. CM2 was expanded in HCM relative to controls. Differential expression analysis identified over 2000 upregulated genes and 486 downregulated genes in HCM cardiomyocytes, enriched for pathways involving growth, protein synthesis, metabolism, stress response, immune pathways, and contractile function. Pseudotime analysis positioned CM2 at a later trajectory stage, and genes with altered dynamic expression along pseudotime, differential expression between conditions, and increased network centrality in HCM were prioritized, resulting in 14 candidate disease regulators including FGF12, CREB5, and BDNF.

Fibroblasts also exhibited distinct disease-associated states, with four fibroblast subpopulations identified. FB1 represented a basal state, while FB2 expressed markers of activated fibroblasts (FAP, POSTN, FN1, COL1A1, COL3A1) and was greatly expanded in HCM. Differential expression in fibroblasts revealed enrichment of ECM organization, TGFβ signaling response, protein translation, metabolism, stress response, immune response, and G protein coupled receptor signaling. Trajectory analysis positioned FB2 at the end of the activation continuum, and prioritization of genes based on trajectory, differential expression, and network centrality yielded 28 candidate fibroblast regulators, including AEBP1, RUNX1, MEOX1, and LEF1, which were validated experimentally.

Across immune and vascular lineages, multiple subpopulations were present, including five macrophage states, with vessel-associated macrophages (MAC1/2) and inflammatory macrophages (MAC5) showing altered proportions in HCM. Cell–cell communication analysis revealed increased overall communication strength in HCM compared to control, driven largely by fibroblast outgoing signaling. Neuronal cells exhibited increased incoming communication, whereas failing CM2 cardiomyocytes showed reduced autocrine and paracrine connections. TGFβ signaling showed the largest divergence between HCM and control. Spatial transcriptomics revealed that failing CM2 cardiomyocytes localized near fibrotic regions, whereas CM1 cardiomyocytes and basal fibroblasts were located in non-fibrotic zones. Activated fibroblasts (FB2) and candidate regulators such as AEBP1, RUNX1, and MEOX1 were enriched in fibrotic areas. Fibrotic regions displayed increased ECM remodeling and immune signaling and reduced contraction and energy metabolism. In vitro perturbation experiments confirmed a functional role for AEBP1 in fibroblast activation, suggesting potential translational relevance.

A follow-up study from the same group that produced the single-nucleus dataset by Larson et al. applied spatial transcriptomics to examine regional transcriptomic differences across varying degrees of myocyte disarray in HCM septal tissue [56]. Tissue sections were stained with morphological markers (desmin, fibroblast activator protein, CD45, and nuclear dye) to delineate cardiac structure, and regions of interest (ROIs) were visually identified and categorized according to the degree of myocyte disarray—normal, mild, moderate, or severe.

Differential expression analysis was performed between multiple ROI categories. Comparison of morphologically normal regions in HCM versus normal control myocardium revealed upregulation of genes related to mitochondrial energetics and downregulation of genes involved in interferon signaling. When comparing HCM regions with increasing disarray severity, mitochondrial pathways remained upregulated, whereas extracellular matrix-related genes were relatively reduced in highly disarrayed zones. The number of differentially expressed genes was greater in normal HCM versus control than in normal versus severe disarray within HCM, suggesting that changes in gene expression may be more pronounced between disease and control than across degrees of disarray.

Ligand-receptor analysis was performed by intersecting differentially expressed genes with the CellChat interaction database. In comparisons between control and HCM regions, signaling pathways such as platelet-derived growth factor (PDGF), NOTCH, neurotrophin, junctional adhesion molecule, and CD46 were downregulated, whereas fibronectin, CD99, cadherin, and amyloid precursor protein signaling were upregulated. Within HCM samples, progressive disarray was associated with a further decrease in CD99-related signaling. In contrast to ligand-receptor analyses derived from dissociated single-nucleus data, this spatially resolved approach incorporates anatomical context and physical proximity between cell populations, thereby prioritizing interaction pairs that are more likely to occur in situ.

Deconvolution analysis using single-nucleus reference data estimated cell-type composition within each ROI. Fibroblast proportions increased in parallel with the severity of myocyte disarray, consistent with localized fibrosis. Regions classified as normal or mildly disarrayed in HCM also showed an increased presence of lymphatic endothelial cells compared with controls.

### 5.4. Concordant and Discordant Molecular Themes Across Studies

Despite substantial differences in sequencing modality and analytic pipelines, several molecular themes recur when comparable cell types are examined across independent studies. Cardiomyocytes consistently show remodeling of sarcomeric and contractile programs, with recurrent upregulation of genes involved in actin binding, muscle contraction and sarcomere organization. Multiple studies also identify increased cardiomyocyte heterogeneity in HCM, including the emergence or expansion of fetal-like or stress-associated states expression and positioned later along inferred disease trajectories.

Altered metabolic programs also represent a concordant theme, although their directionality varies by context. Single-cell and single-nucleus studies identify disease-associated modules related to mitochondrial function, ATP metabolism, oxidative phosphorylation, and energy homeostasis, several of which correlate with cardiomyocyte hypertrophy. Spatial transcriptomic analyses further demonstrate regional enrichment of metabolic and mitochondrial pathways in HCM myocardium with various degrees of myocyte disarray. Together, these findings indicate that metabolic reprogramming is a central feature of HCM, but one that is dynamically regulated across cardiomyocyte states, disease stages, and tissue regions.

In contrast, extracellular matrix remodeling and cell–cell communication pathways show discordances across studies. Single-nucleus RNA sequencing study by Larson et al. reports downregulation of multiple ECM genes in fibroblasts and reduced enrichment of ECM-related and TGF-β-responsive pathways, accompanied by a global decrease in inferred ligand-receptor interactions [59]. However, other studies identify upregulation of ECM-associated genes within cardiomyocytes themselves and activation of fibroblast ECM programs within fibrotic regions [55,61]. In addition, Liu et al. also shows increased overall communication strength in HCM tissue, driven largely by fibroblast signaling, despite reduced autocrine and paracrine signaling in failing cardiomyocyte states. Together, these findings indicate that while several core disease pathways are conserved across studies, others are more sensitive to sequencing modality, patient cohort and analytical pipelines.

## 6. Interpreting Single-Cell Omics Data in HCM

Interpretation of single-cell and single-nucleus omics studies in HCM requires consideration of several methodological and biological sources of heterogeneity. First, snRNA-seq and scRNA-seq quantify different transcript pools. Single-nucleus datasets are enriched for nuclear-retained transcripts while single-cell datasets capture predominantly cytoplasmic and some nuclear transcripts. These intrinsic modality differences can produce non-overlapping disease signals even when studying the same tissue type. For example, Lu et al. directly compared integrated single-cell RNA-seq and single-cardiomyocyte datasets and showed that their global gene-expression distributions differed markedly, with higher within-modality correlation than across modalities [61]. Notably, canonical HCM marker genes such as NPPA, NPPB, TPM3, and RTN4 showed prominent upregulation in HCM only in single-cardiomyocyte RNA-seq datasets, while corresponding changes were modest in single-nucleus datasets. These findings indicate that cardiomyocyte remodeling programs may be underrepresented in snRNA-seq data. In addition, Lu et al. also observed that differential gene expression sensitivity was reduced by low sequencing depth. Together, these observations demonstrate that conclusions regarding cell type specific remodeling in HCM are shaped not only by biological differences but also by RNA capture modality and technical resolution.

Second, tissue source and disease stage influence the observed transcriptional landscape. Septal myectomy specimens represent obstructive HCM in an earlier, likely compensated hypertrophic stage. Explanted failing hearts prior to heart transplant used in some studies reflect end-stage non-obstructive disease at the time of transplant, where secondary heart failure remodeling may dominate the transcriptional signature. Findings from these two biological states are not interchangeable.

Third, patient-level composition differs substantially across studies and can shape conclusions. For example, Larson et al. included mainly older genotype-negative North American patients, whereas Liu et al. studied younger genotype-positive Chinese men [55,59]. These differences in age, ancestry, genetic background, and disease penetrance likely contribute to the divergent findings reported across studies. Gene-positive early disease may have distinct activation programs compared with late-onset gene-negative HCM.

Importantly, analytic pipelines vary substantially across studies and influence both the number of reported cell states and the degree of inferred heterogeneity. Decisions made during quality control, including thresholds for filtering low-quality cells, ambient RNA correction, and doublet removal, affect which cells are retained and can alter the representation of rare or stressed populations. Normalization and batch correction choices, particularly whether datasets are integrated across samples or analyzed separately, can change how cells group together. Downstream choices such as the number of dimensions used, clustering resolution, and clustering method further affect how transcriptional variation is represented. The number of identified cardiomyocyte or fibroblast subpopulations is therefore not a fixed biological constant, but partly an analytical outcome.

In many studies, candidate regulators are prioritized based on network connectivity and trajectory position rather than on differential expression alone. These approaches aim to identify genes that may coordinate broader transcriptional programs or act at key transition points during disease progression. However, different ranking strategies emphasize different biological features, and alternative approaches may yield different candidate genes. As a result, regulator prioritization reflects the analytic framework and underlying assumptions of each study and should be interpreted as hypothesis-generating rather than definitive evidence of causality.

Interpretation of spatial transcriptomic data also requires careful consideration of spatial resolution. Most spatial transcriptomic platforms used in HCM studies capture transcripts from multiple cells within each spatial spot, rather than from single cells. As a result, cell type-specific gene expression and ligand-receptor interactions are inferred based on spot-level expression and computational deconvolution rather than directly measured at the single-cell level. This limits certainty regarding the precise cellular sources and targets of inferred signaling interactions. Integration with single-cell or single-nucleus reference datasets and validation using histology or in situ assays can improve interpretation but do not fully eliminate these resolution constraints.

## 7. Translational and Clinical Implications

Single-cell and spatial transcriptomic studies have transformed our understanding of HCM from a monogenic disease of the sarcomere to a heterogeneous disease involving cell type specific remodeling, altered cell–cell communications, and dysregulated transcriptional programs. Such insights set the stage for precision medicine approaches in HCM where therapies can be tailored to a patient’s molecular phenotype in addition to their clinical presentation.

There has been growing interest in precision medicine within cardiovascular disease, and in theory, monogenic cardiomyopathies such as HCM appear to be ideal candidates. However, multiple studies have challenged the traditional notion of HCM as a strictly monogenic disease. Large population-based studies have shown that common variants contribute substantially to HCM risk. Biddinger et al. derived a polygenic risk score (PRS) that was strongly associated with HCM and improved prediction when combined with rare variants and clinical factors [63]. Zheng and colleagues developed and validated a polygenic score that increased disease risk in the general population, modified penetrance among pathogenic variant carriers by nearly ten-fold across score quintiles, stratified risk in relatives, and predicted adverse outcomes [64]. Tadros et al. identified 70 HCM-associated loci through large-scale genome-wide association study and demonstrated that their PRS captured meaningful polygenic contribution to disease susceptibility [65]. Together, these findings indicate that HCM reflects a combination of rare pathogenic variants and polygenic background, suggesting that precision medicine in HCM may be better conceptualized as targeting shared downstream pathological pathways such as fibrosis, metabolic remodeling, or altered signaling networks rather than focusing solely on correction of a single defective gene.

In clinical reality, HCM-related mortality is already remarkably low (≤0.5% per year, with sudden cardiac death ~0.3% per year) under current management strategies. This favorable survival means that the rationale for gene-targeted or molecular therapies must be weighed carefully, especially given uncertainties around long-term efficacy, safety, feasibility, and cost. Translation is also limited by the need to link single-cell molecular states to meaningful clinical outcomes such as arrhythmic risk, heart failure progression, or treatment response. At the same time, HCM continues to carry substantial morbidity, including heart failure symptoms, atrial and ventricular arrhythmias, and frequent healthcare use. These ongoing clinical burdens underscore the need for new therapeutic approaches. Single-cell and spatial omics studies help address this gap by identifying disease-associated pathways and cell states that may serve as future treatment targets.

From a translational perspective, not all molecular axes identified by multi-omics studies are equally suited for therapeutic targeting. Pathways related to sarcomere contractility, metabolic and mitochondrial remodeling, and fibrosis-associated signaling recur across independent cohorts and represent plausible targets for mechanism-based intervention. In contrast, many transcriptional signatures that define specific cardiomyocyte or fibroblast states, trajectory position, or regional histopathologic features are more appropriately viewed as descriptive biomarkers that reflect disease stage, cellular stress, or tissue remodeling rather than direct therapeutic targets.

Conceptually, these data suggest a framework in which conserved pathways may serve as intervention points, while cell state-specific or spatially restricted signatures may inform disease stratification or monitoring. However, current studies vary substantially in how cell states and pathways are defined and prioritized, and none have yet linked these molecular features to patient-level clinical outcomes. As a result, translation of single-cell findings into clinically meaningful endotypes will require standardized analytic approaches, cross-cohort validation, and integration with shared clinical endpoints before pathway-level insights can be reliably mapped to biomarkers, interventions, and outcomes.

## 8. Current Limitations and Challenges

Looking ahead, several considerations will be important for advancing single-cell studies in hypertrophic cardiomyopathy. A key challenge lies in the integration of datasets across studies, as differences in patient characteristics, tissue source, nuclei or cell isolation, sequencing chemistry, and computational pipelines can all influence downstream clustering and interpretation. As a result, variation in the number or identity of subpopulations likely reflects both true biological heterogeneity and technical differences. Developing shared standards for data processing, annotation, and reporting will improve comparability and facilitate the construction of unified HCM reference atlases.

In addition to analytical standardization, greater consistency in tissue sampling and processing will be critical for studies of obstructive HCM. Septal myectomy specimens provide a unique opportunity to study disease-relevant myocardium at an earlier stage, but most studies provide limited detail on the specific septal regions sampled beyond the designation of ‘septal myectomy’ tissue. Future work would benefit from standardized sampling of defined septal regions, with annotation of proximity to areas of fibrosis or myocyte disarray based on histology. Parallel profiling of relatively preserved myocardium and adjacent fibrotic or severely disarrayed regions within the same specimen would allow internal comparison while controlling for patient-level confounders.

Pre-analytical variables should also be reported in a standardized manner, including ischemic time, time to freezing, fixation method, and tissue handling prior to nuclei or cell isolation, as these factors can influence RNA quality and gene detection. Adoption of minimal reporting standards for septal myectomy processing would improve reproducibility and facilitate cross-study integration in obstructive HCM.

Single-cell RNA sequencing itself also has inherent technical limitations. It captures mainly polyadenylated transcripts, missing non-polyA RNAs, intronic and unspliced transcripts, and other regulatory RNA species that may play roles in transcriptional control and chromatin organization. Emerging multimodal approaches such as combined scRNA-seq and scATAC-seq, spatial transcriptomics, and proteomic profiling will provide a more comprehensive view of gene regulation and cell–cell communication within the myocardium. Finally, to translate molecular insights into clinical relevance, findings from single-cell studies must be validated in model systems and linked to patient phenotypes. Prioritized pathways and regulators should be tested in relevant model systems using genetic or pharmacologic perturbation to assess functional effects on hypertrophy, fibrosis, metabolism, and electrophysiology. Spatial transcriptomic findings can be further validated through histology, in situ hybridization, and protein-level assays to confirm region-specific expression and cell–cell interactions. Integrating multi-omics data with patient level imaging, genetic, and clinical information will help connect cellular programs to disease stage, prognosis, and therapeutic response. To date, none of the studies discussed in this review have directly correlated single-cell or spatial molecular signatures with patient-level clinical outcomes. Establishing such correlations is an important next step for the field. Clinically relevant outcomes such as response to myosin inhibitors or other medical therapies, progression to atrial fibrillation or heart failure, arrhythmic risk, and the extent or progression of myocardial fibrosis assessed by imaging or histology are well suited for integration with single-cell and spatial transcriptomic data. Linking cell type-specific transcriptional states or signaling pathways to these outcomes may help identify molecular programs associated with disease progression or treatment response.

## 9. Conclusions

Single-cell and spatial transcriptomic studies have the potential to support precision medicine in hypertrophic cardiomyopathy by revealing shared molecular pathways and cellular states that underlie disease heterogeneity. Across studies, recurrent themes include sarcomeric remodeling, metabolic reprogramming, fibroblast activation, and altered cell–cell communication, highlighting that HCM is a multicellular and spatially organized disease rather than a purely sarcomeric disorder.

Together, these findings suggest several potential axes for stratification, including sarcomere mutation status (genotype-positive versus genotype-negative), transcriptional states associated with different patterns of hypertrophic remodeling, and molecular signatures related to fibrosis, metabolism, or altered cell–cell signaling. Integrating molecular profiles with imaging, hemodynamic measures, and longitudinal clinical data may help identify groups of patients with shared molecular features and differing disease progression or treatment response. These efforts will help bring precision medicine in HCM closer to practice where treatment strategies are informed not only by anatomy and symptoms, but also by the underlying molecular changes driving each patient’s disease.

## Figures and Tables

**Table 1:** Summary of single-cell omics studies using human septal myectomy tissue.

No	Year	Author	HCM Tissue Source	Control Tissue Source	Technique	Cell Type	Validation	Findings
1	2022	Larson	Septum	Explanted septum	Single-nucleus	All	No	snRNA-seq of septal myectomy and control hearts revealed 10 cell types and 34 subpopulations, including 13 cardiomyocyte and 8 fibroblast states. HCM showed upregulation of sarcomeric and down regulation of ECM genes, global loss of intercellular communication, but selective gain of integrin-mediated fibroblast-cardiomyocyte signaling.
2	2022	Wehren	Septum	Explanted septum and LV (from previous studies)	Single-cell	CM	Ex vivo	scRNA-seq of isolated cardiomyocytes identified six subpopulations with distinct sarcomeric, metabolic, and stress-response programs. NPPA expression defined a fibrotic-border subpopulation; SCENIC and co-expression analyses uncovered regulons linked to sarcomere remodeling, metabolism, and hypertrophy-associated modules correlated with cell size.
3	2023	Lu	Septum	Explanted septum	Single-cell	CM	Ex vivo	scRNA-seq of septal cardiomyocytes identified 191 up/downregulated genes, showing enhanced contractile and developmental programs but reduced oxidative phosphorylation and ribosome biogenesis, consistent with a fetal-like metabolic shift. Meta-analysis revealed conserved structural, calcium handling and MAPK signaling modules. Five distinct cardiomyocyte clusters and increased ECM gene expression in cardiomyocytes.
4	2023	Chen	Septum	Explanted LV (from previous studies)	Single-cell	CM	Ex vivo, in vivo	scRNA-seq of cardiomyocytes identified three subpopulations and five gene modules linked to metabolism, sarcomere structure, and ATP production. The ATP metabolism module correlated with hypertrophy; COX7B emerged as a key Complex IV gene upregulated in early hypertrophy and downregulated in failing hearts, validated in pressure-overload mice.
5	2023	Liu	Septum	Explanted septum	Single-nucleus + spatial transcriptomics	All	In vitro	Integrated snRNA-seq and spatial transcriptomics defined lineage-specific remodeling in HCM, with expansion of vascular lineages and loss of cardiomyocytes and fibroblasts. CM2 (fetal-like) and FB2 (activated fibroblast) states predominated in fibrotic regions. Key regulators (FGF12, CREB5, BDNF, AEBP1, RUNX1) were prioritized; TGFβ signaling showed the largest network divergence.
6	2023	Laird	Septum	Explanted septum	Single-nucleus + spatial transcriptomics	All	No	Spatial transcriptomics mapped transcriptomic gradients across myocyte disarray severity. HCM regions showed mitochondrial upregulation and interferon/ECM downregulation, with altered ligand-receptor pathways (loss of PDGF/NOTCH, gain of fibronectin/CD99). Deconvolution revealed increased fibroblast abundance with worsening disarray.

## Data Availability

No new data were created or analyzed in this study. Data sharing is not applicable to this article.
